# Critical increase in Na-doping facilitates acceptor band movements that yields ~180 meV shallow hole conduction in ZnO bulk crystals

**DOI:** 10.1038/srep44196

**Published:** 2017-03-08

**Authors:** Narendra S. Parmar, Haena Yim, Ji-Won Choi

**Affiliations:** 1 Center for Electronic Materials, Korea Institute of Science and Technology, Seoul 136-791, Republic of Korea

## Abstract

Stable *p*-type conduction in ZnO has been a long time obstacle in utilizing its full potential such as in opto-electronic devices. We designed a unique experimental set-up in the laboratory for high Na-doping by thermal diffusion in the bulk ZnO single crystals. SIMS measurement shows that Na concentration increases by 3 orders of magnitude, to ~3 × 10^20^ cm^−3^ as doping temperature increases to 1200 °C. Electronic infrared absorption was measured for Na-acceptors. Absorption bands were observed near (0.20–0.24) eV. Absorption bands blue shifted by 0.04 eV when doped at 1200 °C giving rise to shallow acceptor level. Na_Zn_ band movements as a function of doping temperature are also seen in Photoluminescence emission (PL), Photoluminescence excitation (PLE) and UV-Vis transmission measurements. Variable temperature Hall measurements show stable *p*-type conduction with hole binding energy ~0.18 eV in ZnO samples that were Na-doped at 1200 °C.

Invention of GaN based blue light-emitting diode (LED)^1^ has benefitted the mankind as it has been used for making short-wavelength LEDs and lasers diodes (LDs). GaN success has fueled the search for the other cheap and environment friendly potential materials as the demands of illumination, and digital information storage is rapidly increasing. ZnO, with a direct band gap of 3.37 eV at room temperature (RT), attracts considerable attention because of its promising applications for blue-UV LED, diode lasers and spintronics^2^,^3^,^4^,^5^,^6^,^7^. In ZnO the free exciton binding energy is 0.060 eV, which makes the excitons stable at RT^8^. ZnO bandgap can also be tuned from 3 eV to 4.5 eV by Cd and Mg alloying, respectively^9^. ZnO turns red by annealing in Zn or Ti environment and the reason has been attributed to oxygen vacancies and hydrogen complexes^10^,^11^,^12^. ZnO has a broad green luminescence band and scientists have no consensus on its origin^13^,^14^. *N*-type conductivity in ZnO has been due to the impurities, point defects and hydrogen as unintentional donor^15^,^16^. *N*-type ZnO doping can be done easily but stable and reliable *p-*type doping has been a challenge^17^,^18^. Look *et al*.^19^, and Tsukazaki *et al*.^20^, reported shallow *p*-type conduction and *p-n* junction devices in nitrogen doped ZnO thin films but the realization of ZnO based *p-n* devices could not be achieved in last 10–15 years. This cast doubt over the stability and the reproducibility of the most promised nitrogen as a shallow acceptor in ZnO. Recently Lyons *et al*.^21^, showed by the first-principles calculations that nitrogen is a deep acceptor, with an high ionization energy of 1.3 eV and can’t lead to *p*-type conduction in ZnO. Tarun *et al*.^22^, experimentally showed similar results in number of bulk ZnO crystals and found that nitrogen is too deep acceptor. There are a number of other reports on *p*-type ZnO and *p-n* devices^23^,^24^,^25^ however, reliability of *p*-type ZnO remains controversial^26^,^27^. It seems, somehow acceptor doping in ZnO thin films could not produce a stable, shallow and reproducible *p*-type conduction and now *p*-type doping in bulk ZnO has to be explored aggressively. *P*-type doping in bulk ZnO can be realized during the growth or by post processing. Some other interesting *p*-type oxide semiconductors have been reported^28^,^29^,^30^ recently with a possibility of wide range of applications. However, they can’t be useful for UV/blue light related emission due to the unsuitability of the band gap. Recent, developments on nano scale ZnO also seem very promising and can potentially impact opto-electronic industry^31^,^32^,^33^,^34^,^35^,^36^,^37^,^38^.

Meyer *et al*.^39^ showed that Na, incorporated by thermal diffusion can also result in relatively shallow acceptors. They carried Na-diffusion by using salts (Na_2_CO_3_, NaOH and Li_3_N) at <900 °C in the bulk ZnO crystals and Na-solubility was ~10^17^ cm^−3^. Recently Parmar *et al*.^40^ showed the substitutional Na doping in the bulk ZnO crystals using positron annihilation spectroscopy that gave Na_Zn_ broad level PL emission peak at ~0.27 eV. Na-doping was done at 900 °C in air using Na_2_CO_3_ salt. Na concentration and RT resistivity was ~(1–3.5) × 10^17^ cm^−3^ and (10^4^–10^5^) ohm-cm, respectively in these Na-doped ZnO crystals. Hall measurement was not possible due to the high resistivity and low Hall voltage on these ZnO crystals. Few other groups have reported their experimental work and first-principles calculations for alkali metals doping (Li, Na, and K) in ZnO^41^,^42^,^43^,^44^. Park *et al*.^45^ calculation found substitutional Na in ZnO has ~0.17 eV ionization energy. Du and Zhang^46^, using hybrid density-functional calculations, found Na-acceptor levels ~0.30 eV. But no studies have been reported on increasing Na-acceptor concentration and their correlation to Na-acceptor level in ZnO crystals. The increase in Na-acceptor concentration was required to realize *p*-type bulk ZnO crystals. Here we report Na-doping at high temperature (1000 °C −1200 °C) under vacuum environment using Na-metal source. This doping method leads to the increase up to ~3 orders of magnitude (~3 × 10^20^ cm^−3^) in Na-acceptor concentration. Such a high doping concentration lowers the hole activation energy by moving Na-acceptor level close to the valence band and thus achieving shallow *p*-type Hall conduction in ZnO crystals.

## Experimental Methods and Results

### Na-doping

Melt grown ZnO bulk single crystals (10 mm × 10 mm × 0.5 mm) were used for Na-doping and Na-metal (Alfa Aeser, 99.95%) was used as a doping source. Na-doping was carried in a laboratory built HV chamber for doping purposes, with in housed HV button heater along with two K-type thermocouples. One thermocouple was near the Na-source while the other one was on the heater. Na-metal was placed in an Alumina boat and ZnO crystal was mounted on the heater (Supplementary Figure S1). The position of the heater and Na-metal was aligned vertically. The distance between the heater and the Na-source was optimized to achieve the average temperature of ~(70–90)°C near Na-source, while the heater was at 1000 °C–1200 °C. The heater was mounted on a manipulator to have the freedom to move it vertically and horizontally to the desired distance from Na-source. The measured optimized distance was 4”– 6” for Na-doping temperatures ranges. This unique experimental set up with the single heater was designed to evaporate Na and dope ZnO, simultaneously. The doping chamber was evacuated to ~(10^−4^–10^−5^) torr and the vacuum valve of the chamber was closed prior to the start of Na-doping. Na-doping was done at 1000 °C, 1100 °C, and 1200 °C on ZnO crystals for ~24 hours each.

### Secondary-ion mass spectrometry

**Figure 1:**
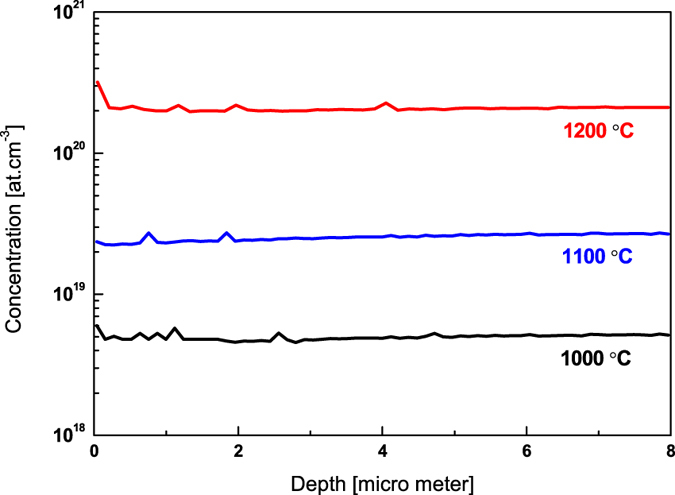
Secondary ion mass spectroscopy (SIMS) of Na-doped ZnO crystals. SIMS depth profiles suggest Na concentration increased >10^20^ cm^−3^ after doping at 1200 °C. Na was diffused in excess of 8 micro meter deep in the bulk ZnO crystals.

Secondary-ion mass spectrometry (SIMS) was performed to examine the incorporation and concentration of Na-dopants. SIMS depth profile measurements of Na-doped ZnO crystals are shown in Fig. 1. These SIMS measurements show the depth profile of Na in excess of 8 microns and with Na concentration ~1 × 10^20^ cm^−3^ when doping was done at 1200 °C. SIMS Na-detection limit was >5 × 10^15^ atoms cm^−3^ and Na was below the detection limit in the control ZnO crystal (annealed at 1200 °C in vacuum).

### Photoluminescence Measurements

**Figure 2:**
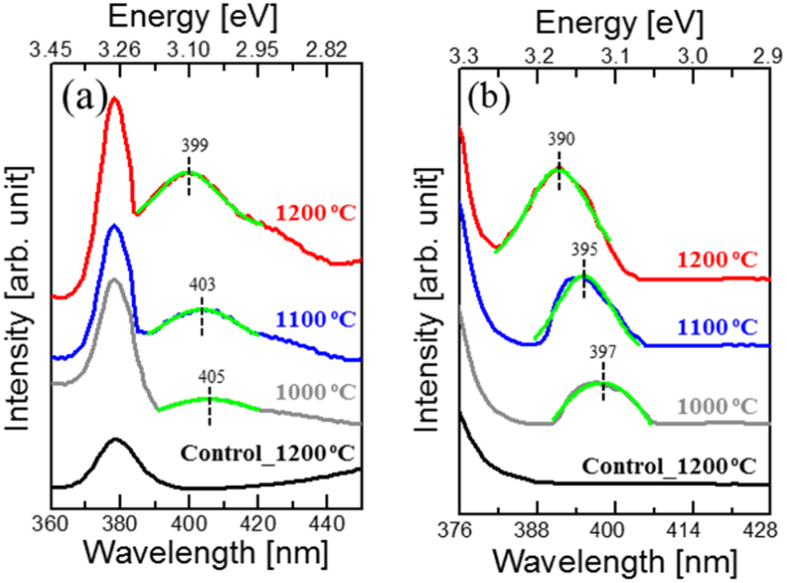
Room temperature (RT) Photoluminescence emission (PL) and excitation (PLE) measurements (**a**) PL emission, showing emergence of donor acceptor pair (DAP) emission after Na-doping **(b)** PLE spectra for 525 nm emission for control and Na-doped ZnO crystals at 1000 °C, 1100 °C and 1200 °C respectively. Na_Zn_ absorption band blue shifts by ~0.08 eV as Na-doping concentration increases to ~10^20^ cm^−3^. Na-acceptor related (DAP) peaks were fitted by single Gaussian model as shown in green color. Data are moved vertically for the clarity.

Photoluminescence emission (PL) spectra were collected at RT using excitation wavelength of 325 nm. Broad PL emission was collected in Na-doped samples with the peak intensity at 400–406 nm region. High Na-doping leads to the blue shift of Na-bands by ~0.05 eV. These PL emission peaks were not observed in the control or Na-doped at 900 °C samples^25^. To further investigate the electronic state of the Na_Zn_ defects, the PL excitation (PLE) technique for 525 nm emission was employed (Fig. 2a). For ZnO crystal that was Na-doped at 1000 °C, the PLE spectrum with a peak at 3.12 eV (397 nm) was observed, (Fig. 2b), confirming a new defect band that acts as a source for 525 nm emission. The control sample does not have this PLE emission while, Na-doped at 900 °C has it at ~3.10 eV. For Na-doped ZnO crystals at 1100 °C and 1200 °C, the PLE peaks were observed at 3.14 eV and 3.17 eV, respectively. This shows that after Na-doping at 1200 °C the Na_Zn_ absorption band was blue shifted by ~0.07 eV giving rise to shallow ~(0.17–0.20) eV Na-acceptor level.

### UV-Vis Transmission Measurements

UV-Vis transmission data at RT were collected in Na-doped ZnO crystals (Supplementary Figure S2). No Na_Zn_ band absorption was seen when ZnO crystal was doped at 900 °C, this could be due to low Na-acceptor concentration. Na_Zn_ absorption bands were observed when Na-doping was done at 1000 °C, 1100 °C and 1200 °C. This could be due to the critical increase in Na-acceptor concentration leading to acceptor band formation. UV-Vis transmission data shows Na_Zn_ absorption bands blue shift by ~0.07 eV after doping at 1200 °C.

For a comparison, as-received ZnO sample was annealed at 1200 °C for 24 hours, with the same experimental conditions, without Na-source. Na_Zn_ related absorption bands were not seen in PL, PLE and UV-Vis measurements. This confirms that observed bands are not the annealing effect but are the consequence of Na-doping.

### Infrared Spectroscopy

**Figure 3:**
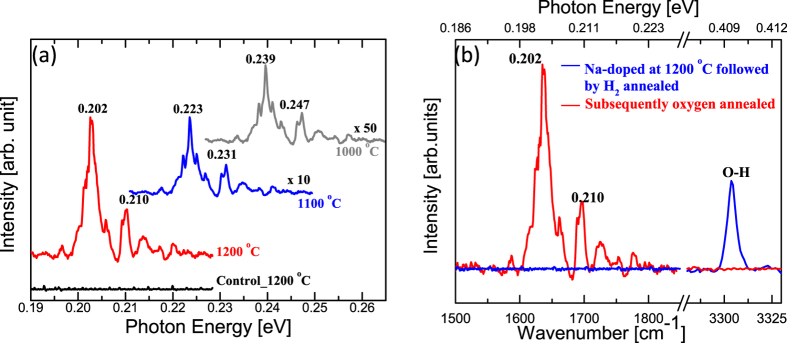
Fourier transform Infrared spectroscopy (FTIR) measurements (**a**) IR absorption bands are shown after Na-doping in ZnO crystals. Absorption band movements are seen towards the low energy (valence band) as Na-doping concentration increases, data are moved vertically for the clarity. The intensity of IR absorption peaks increases with Na-acceptor concentration. (**b**) LVM at 3304 cm^−1^ is seen in Na-doped (1200 °C) and hydrogenated sample, which vanished after oxygen annealing at 900 °C. Na-acceptors were activated after oxygen annealing which gives rise to Na-acceptor related absorption bands at ~(0.195–0.22) eV.

Low temperature (10 K) Infrared transmission measurements were done to acceptors related transitions. Series of IR absorption peaks were seen in Na-doped samples in ~(0.20–0.26) eV range (Fig. 3a). IR absorption bands show shift towards the lower energy as Na-doping concentration increases.

To confirm, these IR absorption are not hydrogen related, a Na-doped (at 1200 °C) sample was annealed at 500 °C in a sealed silica ampoule that was filled with 500 torr H_2_ gas prior to sealing. The annealing was performed in a horizontal tube furnace for 60 hours. In the hydrogenated sample, an O–H local vibrational mode (LVM) was observed at 3304 cm^−1^ at a temperature of 9 K (Fig. 3b) as previously reported by Parmar *et al*.^14^, while no absorption peaks were seen in (0.20–0.26) eV range. The hydrogenated sample turned semi-insulating and Hall measurement could not be done. This hydrogenated sample was oxygen annealed at 900 °C for 45 min. After oxygen annealing, IR absorption bands (0.20–0.26) eV appeared, while the LVM at 3304 cm^−1^ disappeared and Hall measurements achieved *p*-type conduction. This confirms Na-acceptors were compensated by hydrogen annealing giving rise to 3304 cm^−1^ LVM peak. After oxygen annealing, hydrogen was diffused out that activated Na-acceptors^14^ and IR absorption bands (0.20–0.26) eV emerged. Acceptor activation is also required in Mg-doped GaN thin films as Mg-acceptors are compensated by hydrogen during growth^47^.

### Hall Measurements

**Figure 4:**
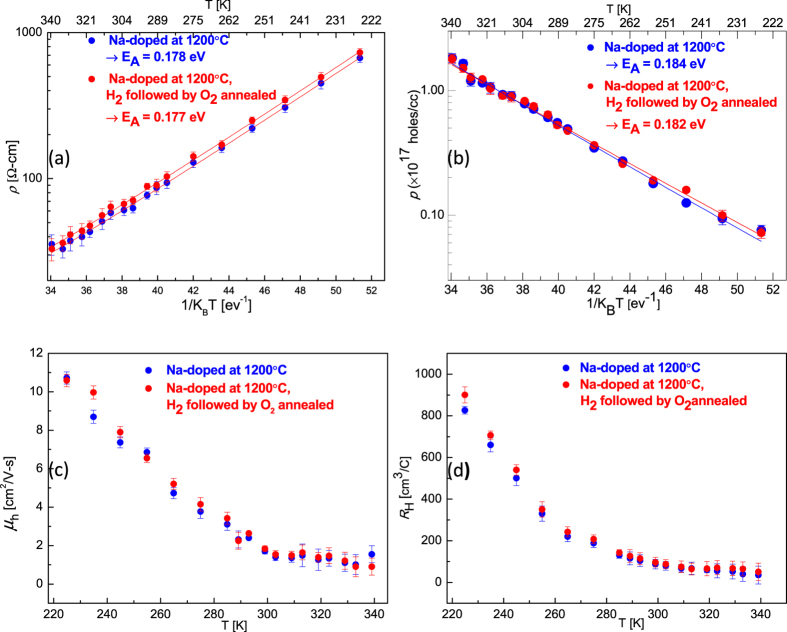
Variable temperature Hall measurements data for Na-doped at 1200 °C and Na-doped at 1200 °C followed by H_2_ and subsequently O_2_ annealed samples (**a**) resistivity (log *ρ*) vs *1/k*_*B*_*T* (**b**) hole density (log *p)* vs *1/k*_*B*_*T* (**c**) hole mobility (*μ*_*h*_) vs *T* (**d**) temperature dependent hall coefficient (*R*_*H*_). The Hall coefficient *R*_*H*_ was positive at all temperatures range (220 < T < 340 K), confirming *p* -type conduction. Arrhenius fit is done to estimate Na-acceptor hole binding energy.

Variable temperature Hall effect measurements were done using Van der Pauw method. For electrical measurements, Ohmic contacts were made on Na-doped samples using MoO_2_^25^. At RT the control ZnO crystal has <1 ohm-cm resistivity, electron density ~(5–6) × 10^16^ cm^−3^ and mobility ~190 cm^2^/V-s, after Na-doping at 1000 °C the resistivity increased to 8000 ohm-cm. The RT resistivity decreased to ~2100 ohm-cm and ~(60–70) ohm-cm after Na-doping at 1100 °C and 1200 °C, respectively. The decrease in RT resistivity is consistent with the increase in Na-acceptor concentration as the doping temperature increases. The majority charge carrier concentration on Na-doped samples at 1000 °C and 1100 °C could not be determined as Hall measurements were not conclusive. However, Na-doped at 1200 °C samples show stable *p*-type conduction and variable temperature Arrhenius fit gives hole activation energy ~0.184 eV. Na-doped (1200 °C) sample that was hydrogenated and subsequently oxygen annealed at 900 °C also achieves *p*-type conduction with ~0.182 eV hole binding energy. The observed hole mobility in these Na-doped ZnO crystals was ~2 cm^2^/V-s at RT (Fig. 4).

## Discussion

Heavy doping modifies the electronic properties of a semiconductor are commonly understood in terms of band tailing and of a reduction of the fundamental energy gap. Band tailing is a result of the random nature of the impurity distribution and the band gap shrinkage represents the self-energy of the various interactions of the charge carriers. In Na-doped samples no band tailing or shrinkage effect was seen as band edge and bandgap remains intact as seen in optical transmission measurements.

The ionization energy of an acceptor atom may be estimated by assuming hydrogenic model of the acceptor,


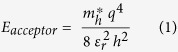


where, 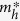 is the hole effective mass, *ε*_*r*_ is the relative permittivity of ZnO, *h* is Planck’s constant and *q* is the electronic charge. Optical measurements shows large (0.04–0.07) eV blue shifts in Na-acceptor level. The favorable change in 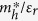 will decrease the hole binding energy. Very high Na-doping can also broaden the acceptor level to form an impurity band due to the wave functions overlap. Broadening of Na-acceptor level is seen in all optical measurements (Supplementary Table S1), Na-acceptor band can be formed at such a high doping concentration which can result in the decrease in Na-acceptor hole binding energy as observed in optical and electrical measurements in number of samples. However, the increase in the relative permittivity (*ε*_*r*_) of ZnO crystals after heavy Na-doping can’t be ruled out completely, which can also contribute in the decrease of Na-acceptor energy level.

Na-doped at 1200 °C sample showed two major IR absorption peaks at 0.203 eV and 0.210 eV followed by continuum absorption. These peaks were not present in the control sample. We attribute the peaks to electronic transitions of neutral Na-acceptors. Such transitions can be modeled by a single-hole hydrogenic model. The dip at 0.208 eV and 0.218 eV is due to multiphonon absorption (LO + nTA). Na-doped at 1000 °C and 1100 °C samples also show two major IR absorption peaks at (0.233, 0.231) eV and (0.239, 0.247)eV, respectively and multiphonon absorption dips followed by continuum absorption. The major IR absorption peaks at (0.239 eV) is blue shifted by 0.037 eV, when doped at 1200 °C (Fig. 3). The intensity of IR absorption peaks increases with Na-doping which is expected due to the increase in Na-acceptor concentration rendering more hole in the valence band. Assuming Na-doping concentration ~1 × 10^20^ cm^−3^ as measured by SIMS, with ~(0.18–0.19) hole energy, ionization of ~(7.4–5) × 10^−2^ % of Na-acceptors will occur, which is equivalent to ~(7.4–5) × 10^16^ holes.cm^−3^ at RT. Hall measurement shows (4.5–5.5) × 10^16^ holes.cm^−3^ at RT. The observed hole concentration is close to the theoretical prediction of ionized population by Fermi-Dirac statistics. Prior to Na-doping, samples were *n*-type having electron concentration ~(5–6) × 10^16^ cm^−3^ at RT with the activation energy ~0.065 eV (Supplementary Figure S3), which leads to ~10^18^ cm^−3^ donor impurity presence. This indicates ~1% of Na-dopants were lost to overcome the *n*-type conduction and to move the Fermi level towards the valence band.

The carrier density was calculated from the Hall coefficient assuming single band conduction, i.e., *p* = 1/*qR*_*H*_. The carrier density increased by a factor of 3.5 from ~5 × 10^16^ holes.cm^−3^ at 290 K to ~1.8 × 10^17^ holes.cm^−3^ at 340 K. Assuming the model of thermal activation of carriers from defect to band states is valid, then the hole carrier density is given by:


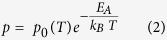


where, *p*_0_ is temperature dependent prefactor, *E*_*A*_ is the acceptor activation energy relative to the valence band edge and *k*_*B*_ is Boltzmann’s constant. Data appear linear on a log *p* vs *1/k*_*B*_*T* plot and ignoring the prefactor temperature dependency, we obtain the slope of the line *E*_*A*_ = (0.185–0.190) eV (Fig. 4b). This suggests thermal activation of the holes from defect (Na_Zn_) to valence band states, and subsequent hole conduction in valence band.

The resistivity data also appear linear on a log *ρ* vs *1/T* plot (Fig. 4a). In a band-conduction model, the resistivity is given by Arrhenius-type behavior:


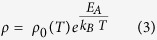


but the prefactor temperature dependency may be significant. Nevertheless, if we ignore the prefactor temperature dependency, we obtain *E*_*A*_ = (0.176–0.178) eV. These values are close to the values *E*_*A*_ = (0.185–0.190) eV, extracted from the carrier-density data, which indicates that temperature dependency of the prefactor in Eq. (2 &3) is sufficiently weak to ignore. Observed hole mobility varied ~(10–1 cm^2^/V-s) in 220 < T < 340 K temperature range (Fig. 4c). Reliable and reproducible results with low contact resistance were limited to in the temperature range (220 < T < 340 K). The Hall coefficient (*R*_*H*_) was positive at all temperatures (Fig. 4d), varying (900 > R_H_ > 35 cm^3^ C^−1^) confirming *p* -type conduction. Na-doped at 1000 °C and 1100 °C samples have much higher resistance which resulted in very low Hall voltage *V*_*H*_ and it was below the detection limit of the hall apparatus and thus definite majority charge carrier could not be determined. Also, if mobility is very low it’s hard to get conclusive Hall measurements^48^. Further, a ZnO crystal was Na-doped at 1200 °C and was subsequently polished for ~20 min to remove any surface effects in IR and electrical measurements. In this polished sample Na-acceptor IR bands (Supplementary Figure S4), and *p*-type Hall conduction (Supplementary Figure S5) were observed, confirming *p*-type behavior is not the surface but the bulk effect.

In conclusion, novel designed setup led the significant increase ~10^20^ cm^−3^ in Na-acceptor concentration when doped at 1200 °C. This resulted in the decrease of Na-acceptor energy to ~(0.18–0.19) eV. Shallow *p*-type Hall conduction was achieved due to the blue shift of Na-acceptor level/band (Supplementary Figure S6) caused by heavy Na-doping. These results can potentially pave a way to get ZnO based optoelectronic devices. Future work includes to further increase the doping temperature and investigate Na_Zn_ acceptor energy band response and Hall conduction.

## Additional Information

**How to cite this article**: Parmar, N. S. *et al*. Critical increase in Na-doping facilitates acceptor band movements that yields ~180meV shallow hole conduction in ZnO bulk crystals. *Sci. Rep.* **7**, 44196; doi: 10.1038/srep44196 (2017).

**Publisher's note:** Springer Nature remains neutral with regard to jurisdictional claims in published maps and institutional affiliations.

## Supplementary Material

Supplementary Information
