# Supplementary material for: Critical increase in Na-doping facilitates acceptor band movements that yields ~180 meV shallow hole conduction in ZnO bulk crystals
Source: Sci Rep. 2017 Mar 8;7:44196. doi: 10.1038/srep44196 (PMC5341155; doi:10.1038/srep44196)
Supplement: Supplementary Information [file srep44196-s1.pdf]

Supporting Information

Critical increase in Na-doping facilitates acceptor band movements that yields ~ 180meV shallow hole conduction in ZnO bulk crystals

Narendra S. Parmar*, Haena Yim and Ji-Won Choi*

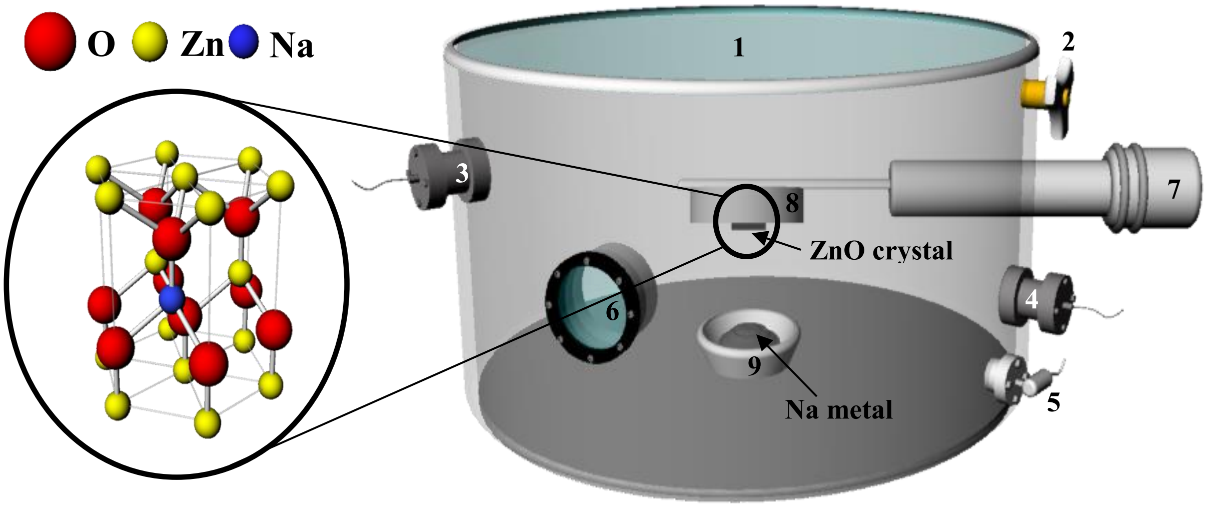

Figure S1. Schematic diagram of Na-doping chamber (1) Chamber's glass lid (2) Chamber's open/close valve (3) Heater feed through (4) Thermocouple feed through (5) To pump connector (6) See through window (7) Manipulator (8) Button heater (9) Na-metal in alumina boat.

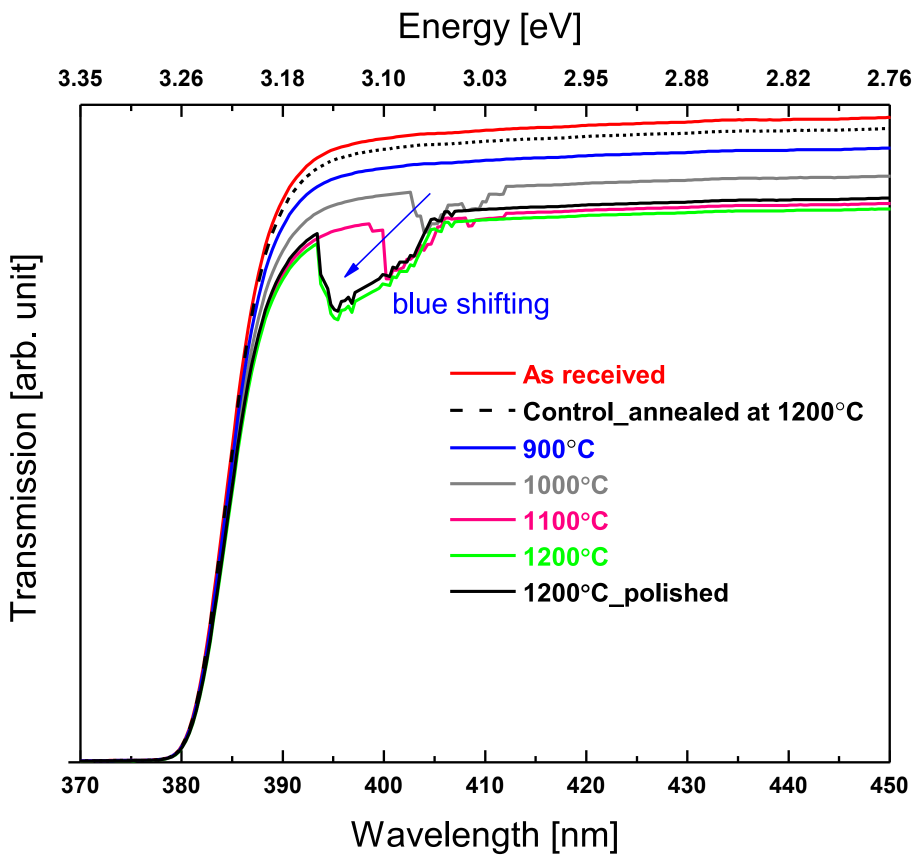

Figure S2. Room temperature UV-Vis transmission spectrum of as-received, control and Na-doped ZnO crystals at 900ºC, 1000ºC, 1100ºC and 1200ºC respectively. Na_{Zn} absorption band blue shifts by ~ 0.07 eV after Na-doping at 1200ºC.

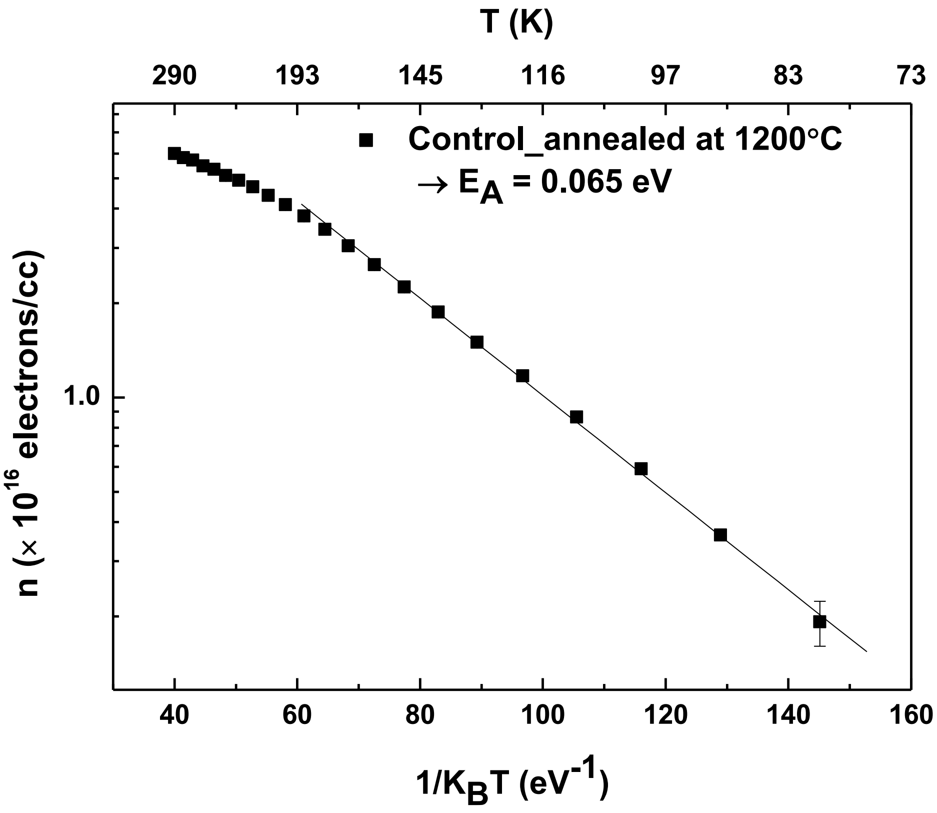

Figure S3. Hall measurement data *n* vs $1/K_BT$ for control ZnO crystal. Arrhenius fit gives electron activation energy ~ 0.065 eV.

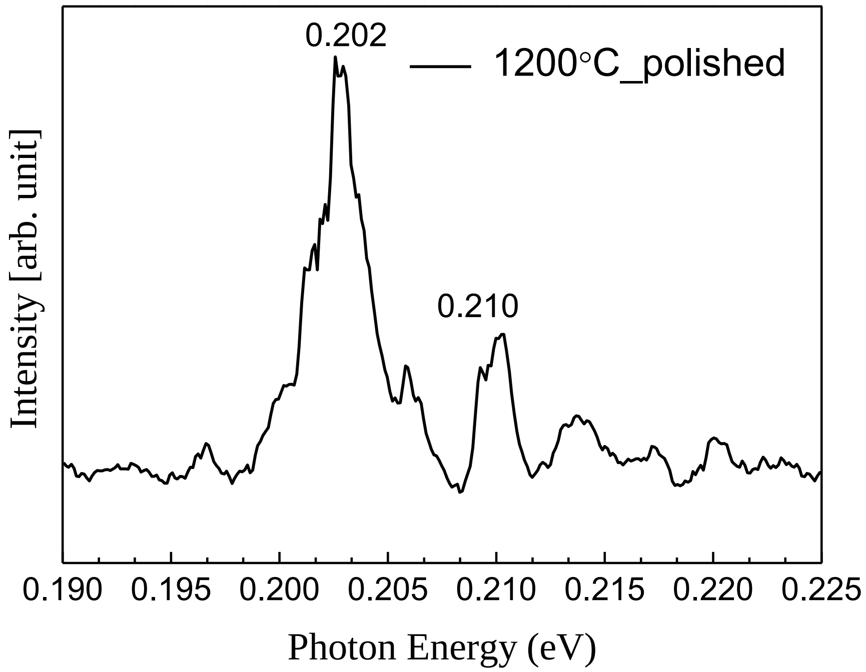

Figure S4. Fourier transform Infrared spectroscopy (FTIR) measurement on Na-doped at 1200C and polished ZnO crystal. Na-acceptor related absorption bands are observed at ~ (0.195 – 0.22) eV.

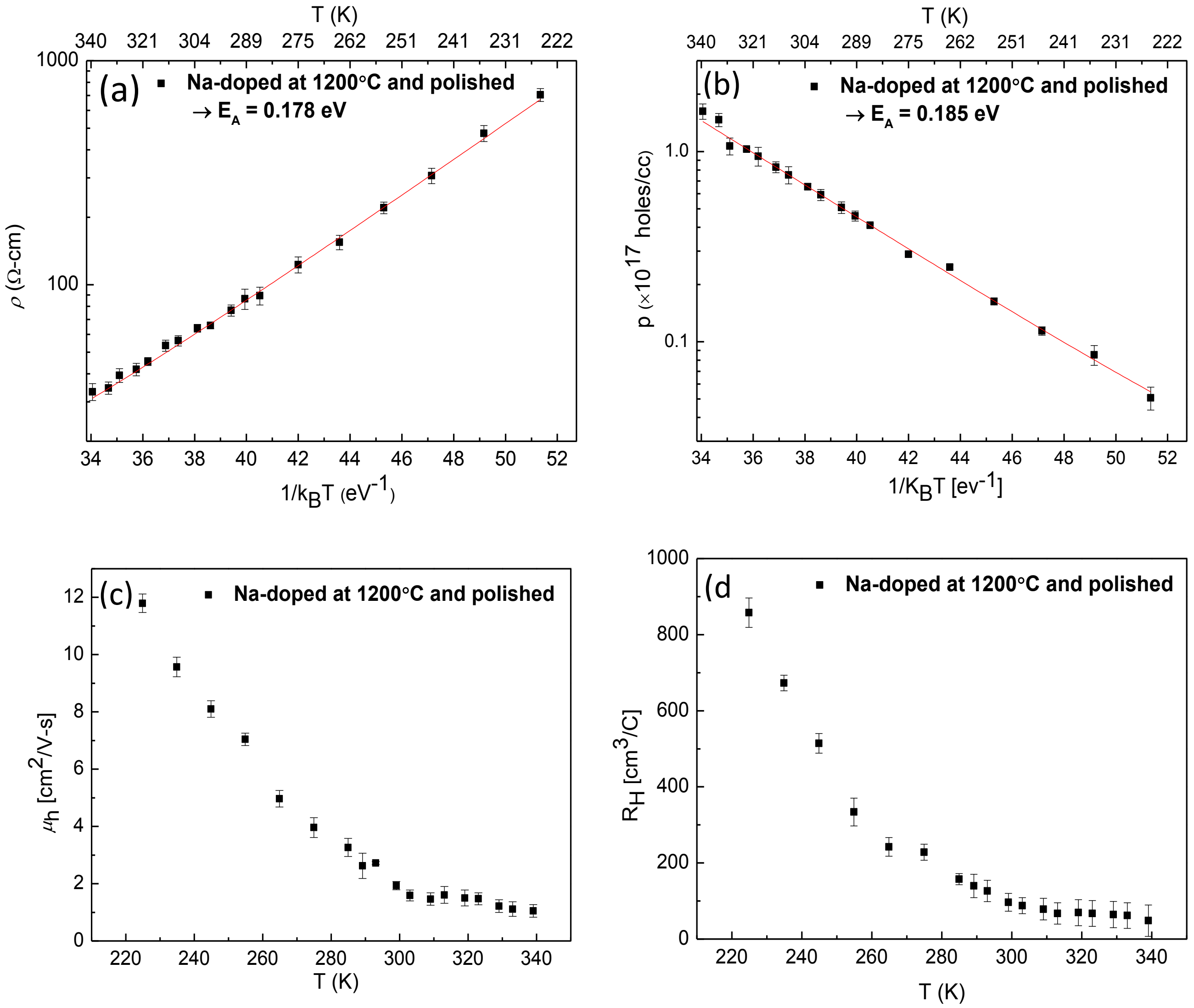

Figure S5. Hall measurement data on Na-doped at 1200ºC and subsequently polished ZnO crystal. (a) resistivity (log ρ) vs $1/k_BT$ (b) hole density (log p) vs $1/k_BT$ (c) hole mobility (μ_h) vs T (d) temperature dependent hall coefficient (R_H). The Hall coefficient R_H was positive at all temperatures range (220< T <340 K), confirming p -type conduction. Arrhenius fit is done to estimate Na-acceptor hole binding energy.

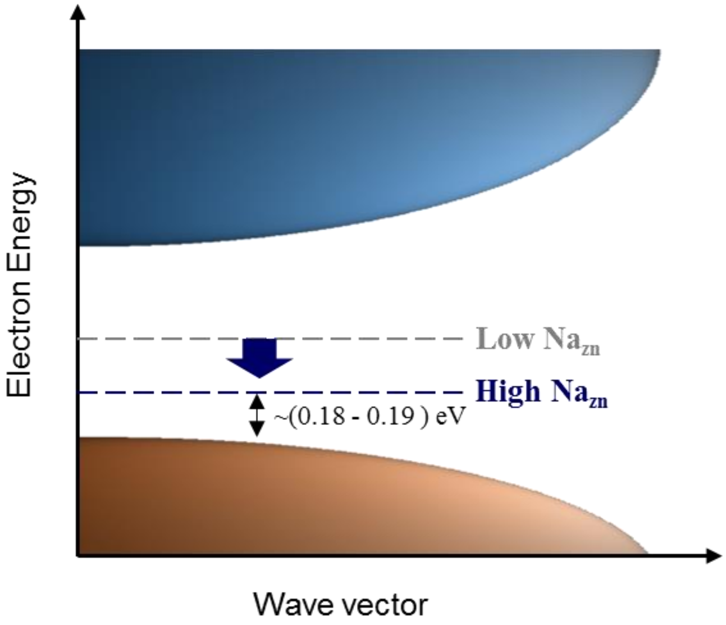

Figure S6. Band schematics for blue shifting of Na-acceptor level after heavy doping.

Doping temperature	Na-acceptor, Gaussian Centroid (eV)		Na_{Zn}: Activation energy (eV)	
	PL	PLE	Hall	PL
1000 °C	3.058 (σ = 0.080)	3.12 (σ = 0.20)	--	0.252
1100 °C	3.074 (σ = 0.085)	3.14 (σ = 0.033)	--	0.236
1200 °C	3.104 (σ = 0.102)	3.17 (σ = 0.039)	0.186 (±0.0053)	0.206

Table S1. PL Na-acceptor band (centroid) shifts towards the valence band along with the band broadening as Na-acceptor concentration increases with temperature. A comparison of Na_{Zn} acceptor energy from Hall and PL is also given.
